# Senescence-Associated Molecules and Tumor-Immune-Interactions as Prognostic Biomarkers in Colorectal Cancer

**DOI:** 10.3389/fmed.2022.865230

**Published:** 2022-04-12

**Authors:** Franziska Kellers, Aurélie Fernandez, Björn Konukiewitz, Mario Schindeldecker, Katrin E. Tagscherer, Achim Heintz, Moritz Jesinghaus, Wilfried Roth, Sebastian Foersch

**Affiliations:** ^1^Institute of Pathology, University Medical Center Mainz, Mainz, Germany; ^2^Institute of Pathology, Christian-Albrecht University of Kiel, Kiel, Germany; ^3^Department of General, Visceral and Vascular Surgery, Catholic Hospital Mainz, Mainz, Germany; ^4^Department of Pathology, University Hospital Marburg, Marburg, Germany

**Keywords:** cellular senescence, colorectal cancer, senescence-associated secretory phenotype (SASP), prognostic biomarker, senolysis

## Abstract

**Background and Aims:**

The initiation of cellular senescence in response to protumorigenic stimuli counteracts malignant progression in (pre)malignant cells. Besides arresting proliferation, cells entering this terminal differentiation state adopt a characteristic senescence-associated secretory phenotype (SASP) which initiates alterations to their microenvironment and effects immunosurveillance of tumorous lesions. However, some effects mediated by senescent cells contribute to disease progression. Currently, the exploration of senescent cells' impact on the tumor microenvironment and the evaluation of senescence as possible target in colorectal cancer (CRC) therapy demand reliable detection of cellular senescence *in vivo*. Therefore, specific immunohistochemical biomarkers are required. Our aim is to analyze the clinical implications of senescence detection in colorectal carcinoma and to investigate the interactions of senescent tumor cells and their immune microenvironment *in vitro* and *in vivo*.

**Methods:**

Senescence was induced in CRC cell lines by low-dose-etoposide treatment and confirmed by Senescence-associated β-galactosidase (SA-β-GAL) staining and fluorescence activated cell sorting (FACS) analysis. Co-cultures of senescent cells and immune cells were established. Multiple cell viability assays, electron microscopy and live cell imaging were conducted. Immunohistochemical (IHC) markers of senescence and immune cell subtypes were studied in a cohort of CRC patients by analyzing a tissue micro array (TMA) and performing digital image analysis. Results were compared to disease-specific survival (DSS) and progression-free survival (PFS).

**Results:**

Varying expression of senescence markers in tumor cells was associated with in- or decreased survival of CRC patients. Proximity analysis of p21-positive senescent tumor cells and cytotoxic T cells revealed a significantly better prognosis for patients in which these cell types have the possibility to directly interact. *In vitro*, NK-92 cells (mimicking natural killer T cells) or TALL-104 cells (mimicking both cytotoxic T cells and natural killer T cells) led to dose-dependent specific cytotoxicity in >75 % of the senescent CRC cells but <20 % of the proliferating control CRC cells. This immune cell-mediated senolysis seems to be facilitated via direct cell-cell contact inducing apoptosis and granule exocytosis.

**Conclusion:**

Counteracting tumorigenesis, cellular senescence is of significant relevance in CRC. We show the dual role of senescence bearing both beneficial and malignancy-promoting potential *in vivo*. Absence as well as exceeding expression of senescence markers are associated with bad prognosis in CRC. The antitumorigenic potential of senescence induction is determined by tumor micromilieu and immune cell-mediated elimination of senescent cells.

## Introduction

Malignant neoplasia of colon and rectum are associated with high morbidity and mortality and account for 10 % of cancer cases and 9.4 % of cancer deaths (1). Molecular mechanisms of colorectal carcinogenesis are increasingly understood, yet the role of cellular senescence and its contribution to survival and treatment outcome of cancer patients remain unclear.

One mechanism in tumor biology that only recently started to gain more attention due to its role in carcinogenesis is cellular senescence. Cellular senescence describes a permanent cell cycle arrest following potentially protumorigenic DNA-damaging incidents in premalignant cells, thereby counteracting malignant progression (2). There is a multitude of trigger mechanisms leading to the initiation of cellular senescence. Eroded telomeres which occur after repetitive cell divisions (3) or cumulative DNA erosions due to sublethal stressful conditions such as oxidative stress (4), proliferative stress due to oncogene-induced mitogenic hyperstimulation (5–7), loss of tumor suppressors (8, 9) or the presence of DNA damaging agents can induce a DNA damage response, arresting the cell cycle of impaired cells (10). Anticancer treatment such as chemotherapeutic agents, ionizing radiation (11–15) as well as targeted therapies are capable of evoking cellular senescence (16–21). Therapy-induced senescence (TIS) has been observed in tumor cells both *in vitro* and *in vivo* (15). Apart from ceasing proliferation, senescent transformation involves characteristic morphological and metabolic changes (22). *In vitro*, senescent cells adopt a characteristic flat, enlarged “fried egg” morphology as well as nuclear alterations (23–26). Increased lysosomal activity, detected by visualization of the lysosomal enzyme Senescence-associated β-galactosidase (SA-β-GAL) at pH 6, is a widely established biomarker of senescent cells (27). While detection of SA-β-GAL may be used for identification of cellular senescence in fresh or frozen cells (28, 29), the enzyme activity-dependent assay cannot be carried out on formalin-fixed, paraffin-embedded (FFPE) tissues (29) and therefore this distinctive feature may not be used to study cellular senescence *in vivo* to a large extent. Due to the irreversible proliferation arrest, the senescent state is strongly associated with an absence of proliferation markers such as Ki-67 and the expression of anti-proliferative proteins (30). The onset of the senescence program involves cell cycle suppressors such as p53, p21, and p16 (22). The extent to which these features are displayed may vary (23) and none of these characteristics are exclusively linked to cellular senescence. Consequently only a combination of markers allows for distinctive identification of senescent cells (31). Recently, there have been approaches to identify novel markers of senescence (32, 33).

Although no longer proliferating, senescent cells remain highly metabolically active and display an altered secretory and signaling activity. Apart from autocrine enforcement of the senescent state, senescent cells induce non-cell-autonomous effects via direct cell-cell contact with nearby cells, paracrine signaling, and secretion of a multitude of factors affecting angiogenesis and immune surveillance of the tissue environment. The SASP, adopted by arrested cells in the presence of DNA impairment, consists of a distinct composition of secreted molecules involving signaling factors like inflammatory cytokines, enzymes and extracellular matrix components (34). The SASP highly depends on the cell type (34) and enables senescent cells to attract immune cells such as macrophages, NK cells and T cells to the site of a tumorous lesion, activating them to specifically eliminate senescent cells and thus promoting the immunosurveillance of the tumor (35, 36). While some senescent cells remain in the tissue for years (29, 37, 38) and eventually contribute to age-related diseases (39, 40), there are settings where the SASP signaling activates an immediate immune response, resulting in the installation of a proinflammatory micromilieu and eventually the removal of the senescent cells (41, 42), termed “senolysis.” This immune cell driven clearance of senescent cells involves the innate (41, 42) as well as the adaptive immune cells (43, 44). There is evidence that senescent cells under senescence surveillance are eliminated by macrophages (45) or NK cell-mediated induction of granule exocytosis (46, 47).

Cellular senescence has been linked to colorectal carcinogenesis (40). The silencing of the senescence-regulating cell cycle suppressors p16 and p53 typically involved in cellular senescence induction (22) is a crucial step to overcome cellular senescence in colorectal carcinogenesis (48, 49). There is first evidence that measurement of cellular senescence might be a predictive parameter in CRC patients (50) but the clinical implications of the contribution of cellular senescence to colorectal carcinogenesis have not yet been studied in a large patient cohort. Since TIS occurs during various CRC therapies, the influence of this biomechanism on disease progression in CRC needs to be investigated in a clinical setting. Furthermore, it might be a promising approach in colorectal cancer therapy to use the potential of the senescence-induced immunosurveillance to counteract malignant progression (51). Evaluating the impact of cellular senescence and the potential of therapy-induced senescence in CRC demands reliable detection methods and biomarkers applicable to FFPE tissue to explore this key mechanism in colorectal carcinogenesis *in vivo*. We further explored the potential of senescence-associated molecules as prognostic and predictive biomarkers in CRC and conducted both *in vitro* and *in vivo* studies to gain a better understanding of the functional role of the interaction between senescent colorectal tumor cells and the immune system.

## Materials and Methods

### Material

A list of antibodies and inhibitors used in this study can be found in Supplementary Table 1.

### CRC Cell Culture

After preliminary experiments with various CRC cell lines, Caco-2 cells were cultivated in MEM (+15 % fetal bovine serum + 1 % pyruvate, 1 % NEAA, 1 % glutamine, 1 % penicillin, 1% streptomycin) at 37°C and 5% CO_2_ on 12-well-plates. Senescence was induced by low dose (5 μM) Etoposide treatment. 24 h after seeding, the growth medium was replaced by medium containing 5 μM Etoposide. Cells treated with equal volumes of Dimethyl Sulfoxide (DMSO) were used as negative control. After 48 h the medium was replaced by growth medium, and cells were allowed to recover. Analyses were performed after 72 h.

Cytoblocks of Etoposide-treated and control cells were generated after harvesting using Accutase (Sigma Aldrich) treatment, formalin (Sigma Aldrich) fixation and embedding in 1 % Agarose. For following analyses, samples were transferred to paraffin and standard sectioning (2 μm) and subsequent staining was performed according to standard protocols used for routine pathology or as published previously (52). Furthermore, transmission electron microscopy (TEM) was performed according to protocols established for routine diagnostics at our institute (53).

### Immune and CRC Cell Culture

NK-92 cells were cultivated in α-MEM + 12.5% fetal bovine serum + 12.5% horse serum + 1% penicillin + 1% streptomycin + 100–200 U/ml IL-2 (48 h)/ 5 ng/ml IL-2 (every 48 h) at 37°C and 5% CO_2._ TALL-104 cells were cultivated in Iscove's Modified Dulbecco's Medium (ATCC) + 20% fetal bovine serum, 2.5 μg /ml human albumin, 0.5 μg/ml D-manitol + 50–100 U/ml recombinant human IL-2 (48 h) at 37°C and 5% CO_2_. For co-culture experiments, Caco-2 cells were cultivated on 6-well-plates and senescence was induced as according to 3.1. After 72 h of recovery, the growth medium was replaced by immune cell growth medium containing immune cells in different target-to-effector ratios. Cells were co-cultivated for up to 180 min. Following 120 min of co-incubation, cells were washed, and non-adherent cells (immune cells and non-vital Caco-2 cells) were removed. The quantity of remaining adherent Caco-2 cells after Co-culture was measured using cell viability assays such as crystal violet (CRV) staining of the remaining adherent cells.

### Senescence and Cell Viability Assays

Cellular senescence was detected by SA-β-Gal staining using the Senescence β-Galactosidase Staining kit according to the manufacturer's instructions (Cell Signaling). In addition, cells were subjected to FACS analysis using the cellular senescence live cell analysis assay (Enzo) and a Becton Dickinson FACScalibur cytometer and Cell Quest Software (BD Bioscience). For viability analysis, Caco-2 cells were treated as described. At the indicated times, cells were washed with PBS, fixed with methanol:ethanol (2:1) and stained with 0.1 % crystal violet for 30 min. The plates were washed in running tap water and air dried for 24 h. Crystal violet was solubilized using 33 % acetic acid for 30 min. The absorbance was measured at 600 nm using a microplate reader (Tecan).

### Live Cell Imaging

Immune cells were added to Etoposide-treated Caco-2 cells as described above. Cells were incubated at 32°C for 180 min. Cell-cell interactions were observed using a Jenoptik GRYPHAX SUBRA camera system in 100 x magnification. Pictures of representative areas were taken with a 30 s interval.

### Patient Cohort

The patient cohort consisted of up to 598 patients diagnosed with primary colorectal carcinoma at the Institute of Pathology of the University Medical Center, Mainz. These patients had not received neoadjuvant treatment prior to their surgery and were treated according to national and WHO guidelines in place at the time. Patients with a hereditary cancer syndrome or history of inflammatory bowel disease were not included in this study. Retrospective use of these and other patients' data as well as material for research purposes was approved by the ethical committee of the medical association of the State of Rhineland-Palatinate [ref. no. 837.075.16 (10394)]. All experiments were in accordance with the Declaration of Helsinki. Characteristics of the patients can be found in Supplementary Table 2.

### Human Tissue Analyses

From each patient, FFPE tissue samples containing tissue of the primary tumor and non-cancerous tissue were obtained from routine procession of the surgery specimens. Clinical data such as age, DSS, PFS, localization and stage of the tumor were obtained. Representative areas of tumor center, invasive margin and non-cancerous epithelium were identified by review of hematoxylin and eosin (H&E)-stained sections from each sample and cores of 1 mm in diameter were obtained using the TMArrayer (Pathology Devices, San Diego, USA) and included in the TMA. From each patient, 3 samples containing representative areas of the primary tumor were included. TMA sections were stained for various senescence-associated molecules and other cell types. Staining of the slides was carried out using an automated staining system (Agilent Technologies) and its respective reagents. IHC-stained TMA sections were digitalised using a Hamamatsu Nanozoomer Series scanner (Hamamatsu Photonics, Hamamatsu, Japan) at 20 x magnification. Slides were thoroughly annotated by a pathology expert, thereby cancerous epithelium and stroma were marked. Digital image analysis was performed using HALO (Indica Labs, Albuquerque, USA). A random forest classifier was trained to discern (cancerous) epithelium and stroma. The percentage of positive cells within the classified tumor cells was obtained. Consecutive sequential TMA sections were co-registered for additional comprehensive morphometric analyses such as distance-measurements.

### Statistical Analyses

Statistical analyses were carried out using GraphPad Prism version 9. Cell viability data was compared with the control group using *t*-test or ordinary one-way ANOVA. Dunnett T3 test (statistical hypothesis testing) was used to correct for multiple testing. For each marker, the values' distribution was analyzed, and cutoff values were chosen to represent meaningful biological groups, while at the same time finding optimal cutoff values. This was done similar to the method proposed by Budczies et al. where “[t]he optimal cutoff is defined as the point with the most significant (log-rank test) split.” These authors have implemented their approach as open source software named the Charité Cutoff Finder (54). Additionally, we also applied the surv_cutpoint capability of the R survminer package which functions in a similar fashion (54). Cutoff values can be found in Supplementary Table 3. Survival analyses were performed using Kaplan-Meier-plots, differences in survival were calculated by performing log-rank Mantel-Cox test.

## Results

**Figure 1:**
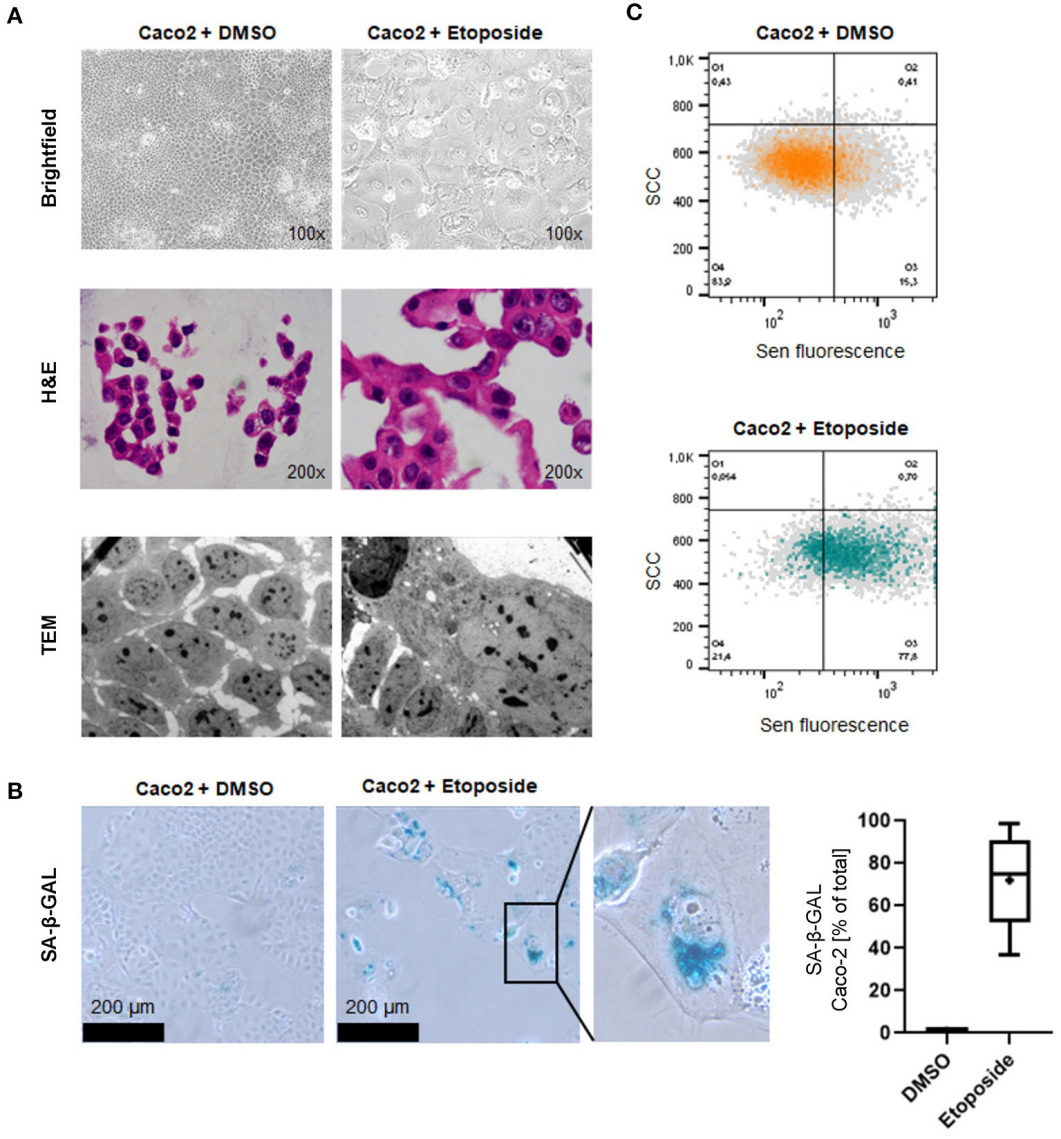
Induction of cellular senescence *in vitro* by Etoposide treatment. **(A)** Morphological appearance of Etoposide (Eto)-treated and control (DMSO) Caco2 cells using brightfield microscopy (upper panel), normal H&E-stained sections of cytoblocks (middle panel) and TEM (lower panel). Senescent cells adopt a characteristic “fried egg” morphology, including an enlarged shape and nucleus. Cytoplasmatic vacuolation is apparent. **(B)** Increased senescence SA-β-GAL staining indicates senescence induction. SA-β-GAL-positive cells were counted after DMSO and Etoposide-treatment. **(C)** FACS analysis confirms senescence induction in Etoposide-treated cells.

Low dose Etoposide treatment induces senescence-related morphological changes and SA-ß-Gal activity. Morphological changes commonly found in senescent cells such as enlarged size and vacuolation could be detected in cells treated with Etoposide using white light microscopy and electron microscopy (Figure 1A). Increased SA-β-GAL activity was observed in 71.9 % of Etoposide-treated Caco-2 cells but only 1.1 % of control cells (*p* < 0.0001**)** (Figure 1B). Senescence induced by Etoposide treatment was also confirmed by FACS analysis (Figure 1C).

**Figure 2:**
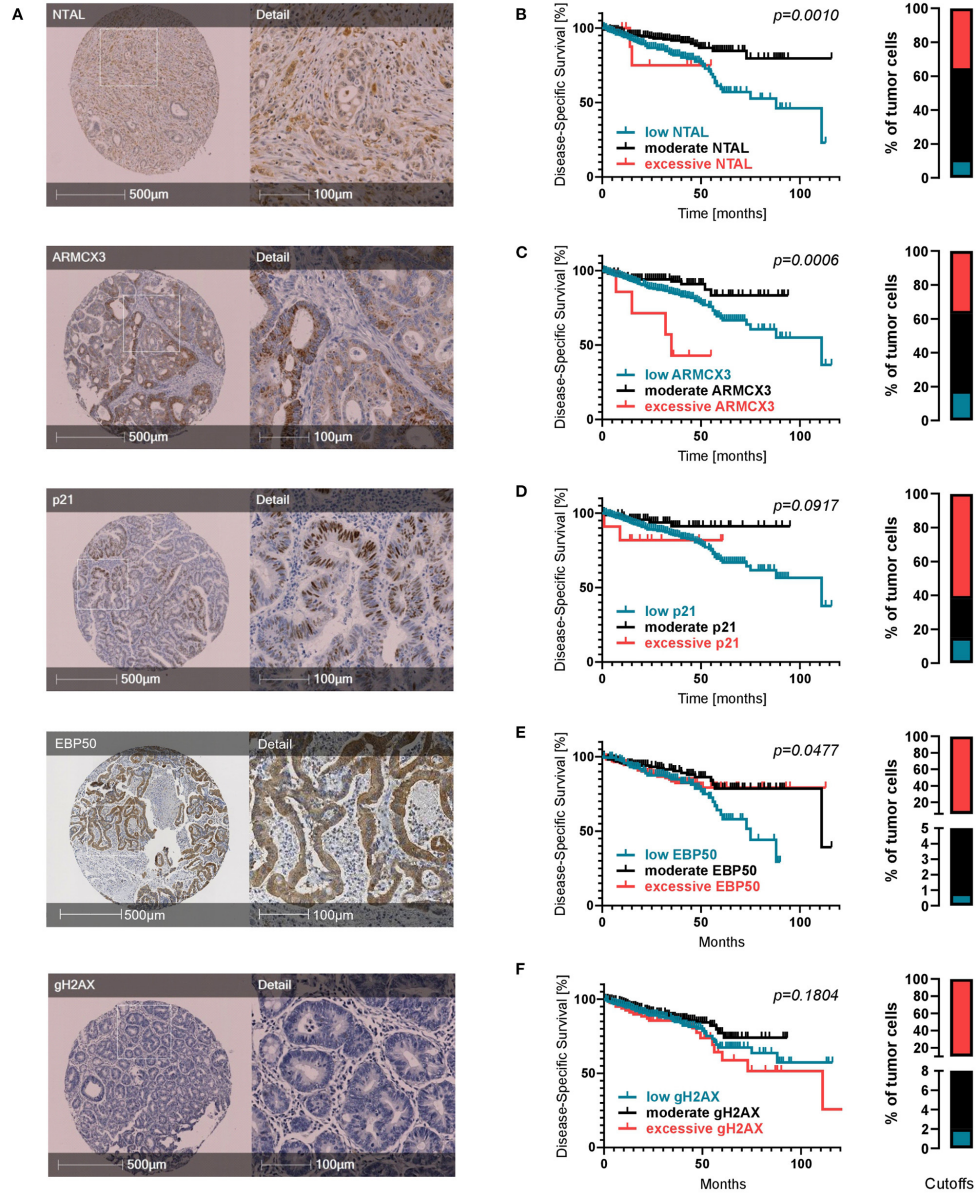
Expression of senescence-associated molecules in CRC patients. **(A)** Representative TMA cores for each IHC marker. **(B–F)** Kaplan-Meier survival analyses regarding expression of senescence markers when divided into three subcohorts: low expression (petrol), moderate expression (black) and excessive expression (red). Cutoffs are displayed as bar graphs on the right of each curve and were calculated using a modification of the Charité Cutoff Finder from (54). Disease-specific survival is shown. Two tier subdivision, progression-free survival and detailed individual cutoff values can be found in the Supplementary Material.

To assess the prognostic potential of senescence-associated molecules suggested by previous studies (32, 33) in a clinical setting, we evaluated various markers in our cohort of CRC patients immunohistochemically (Figure 2A) and observed mixed effects. For NTAL, ARMCX3, p21, and EBP50 the percentage of positive tumor cells showed a statistically significant prognostic effect. High expression of NTAL was linked to a better DSS and PFS (Supplementary Figure 1). A high expression of p21 was linked to a higher PFS (Supplementary Figure 1), underlining the important role of p21-mediated senescence in tumor defense. Evaluating expression of ARMCX3 and EBP50 (Supplementary Figure 1), we found that a high expression was associated with a decreased DSS compared to the group of patients with lower expression levels. This surprising finding led us to try a three-tier cutoff system into excessive, moderate, and low expression (Figures 2B–F, cutoffs on the right and in Supplementary Table 3). Interestingly, for all markers (including gH2AX), using this approach showed that moderate expression was associated with the best prognosis, while both low and excessive expression showed a worse prognosis. This was statistically significant for NTAL, ARMCX3, and EBP50 (Figures 2B–E, Supplementary Figure 1). Taken together, this highlights the dual role of cellular senescence, with both low and excessive expression of senescence-associated markers showing worse DSS and PFS.

**Figure 3:**
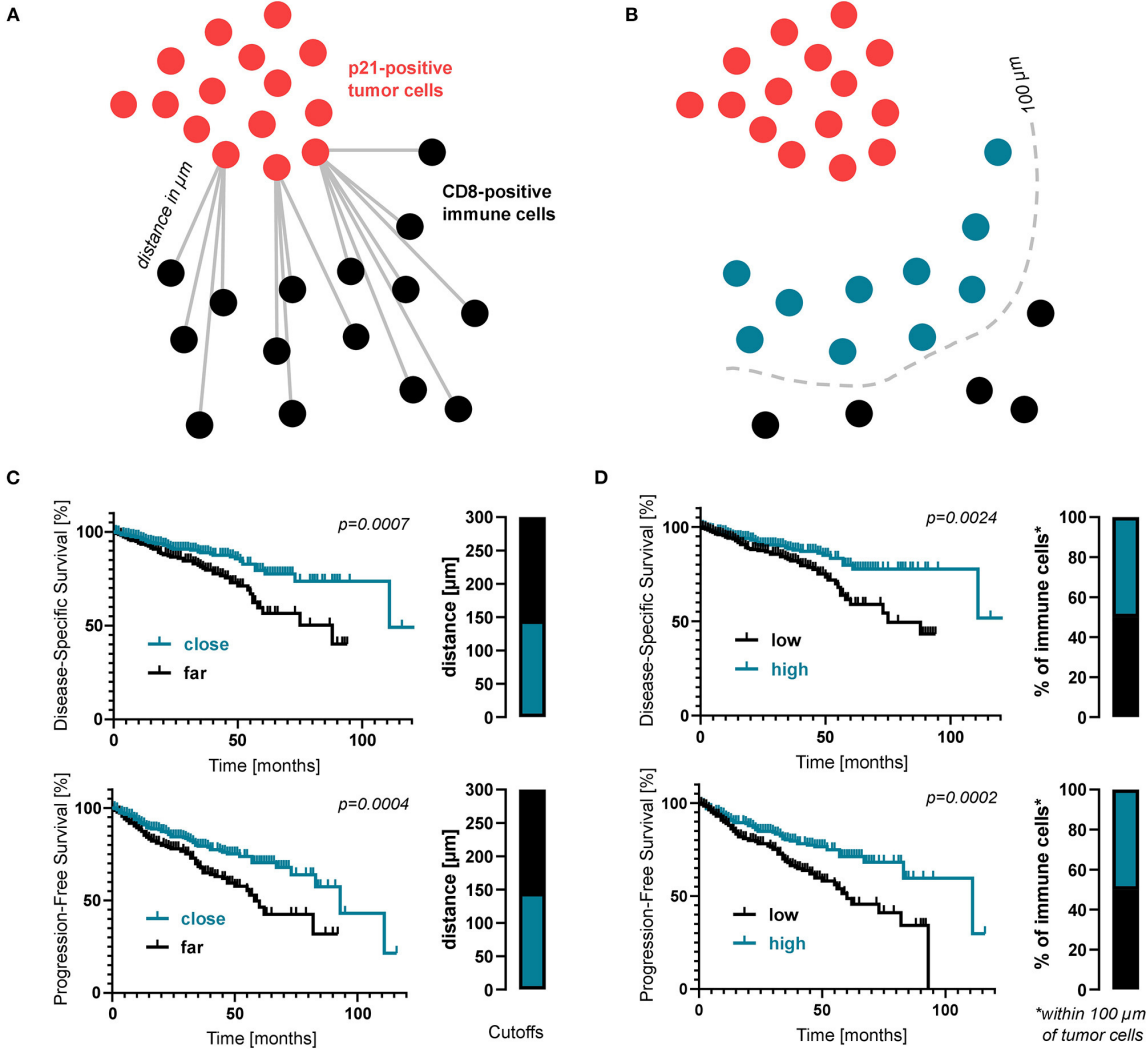
Proximity analysis of senescent cells and immune cells in CRC patients. **(A)** Schematic drawing of how the distance between immune cells and senescent tumor cells was measured. **(B)** Kaplan-Meier survival analyses regarding the average distance between CD8- and p21-positive cells. **(C)** Schematic drawing of how the proportion of immune cells within close proximity (within 100 μm) of senescent tumor cells was measured. **(D)** Kaplan-Meier survival analyses regarding the percentage of CD8-positive cells within 100 μm of p21-positive cells. Again, cutoff values are displayed as bar graphs.

In search of an explanation for this plurivalent effect we hypothesized that the negative prognosis in patients with large numbers of senescent cells might result from a defective interaction between senescent cells and the tumor micromilieu. The excessive numbers of senescent cells in patients with a negative prognosis might reflect accumulation of these cells within the tumor tissue due to an ineffective tumor immunosurveillance and a failure of the immune system to clear of senescent cells. To investigate the immunosurveillance of senescent cells in our clinical cohort and analyse immune cells targeting senescent cells, we visualized the spatial relationship of senescent cells and cytotoxic T cell as stained by CD8 (Figure 3A). Using digital image analysis, consecutive sections with cores of one patient stained for different molecules were co-registered and corresponding tissue areas on the different sections were identified. The average distance between these two cell populations as well as the percentage of CD8-positive cells within 100 μm of p21-positive cells were determined (Figure 3C). To identify a possible impact on survival, proximity data was correlated with DSS and PFS. Interestingly, both a lower average distance between these two cell populations as well as a higher percentage of CD8-postive cells within 100 μm of p21-positive cells were linked to a significantly increased DSS and PFS (Figures 3B,D). This suggests that a closer immunosurveillance of the lesion improves the prognosis of CRC patients.

**Figure 4:**
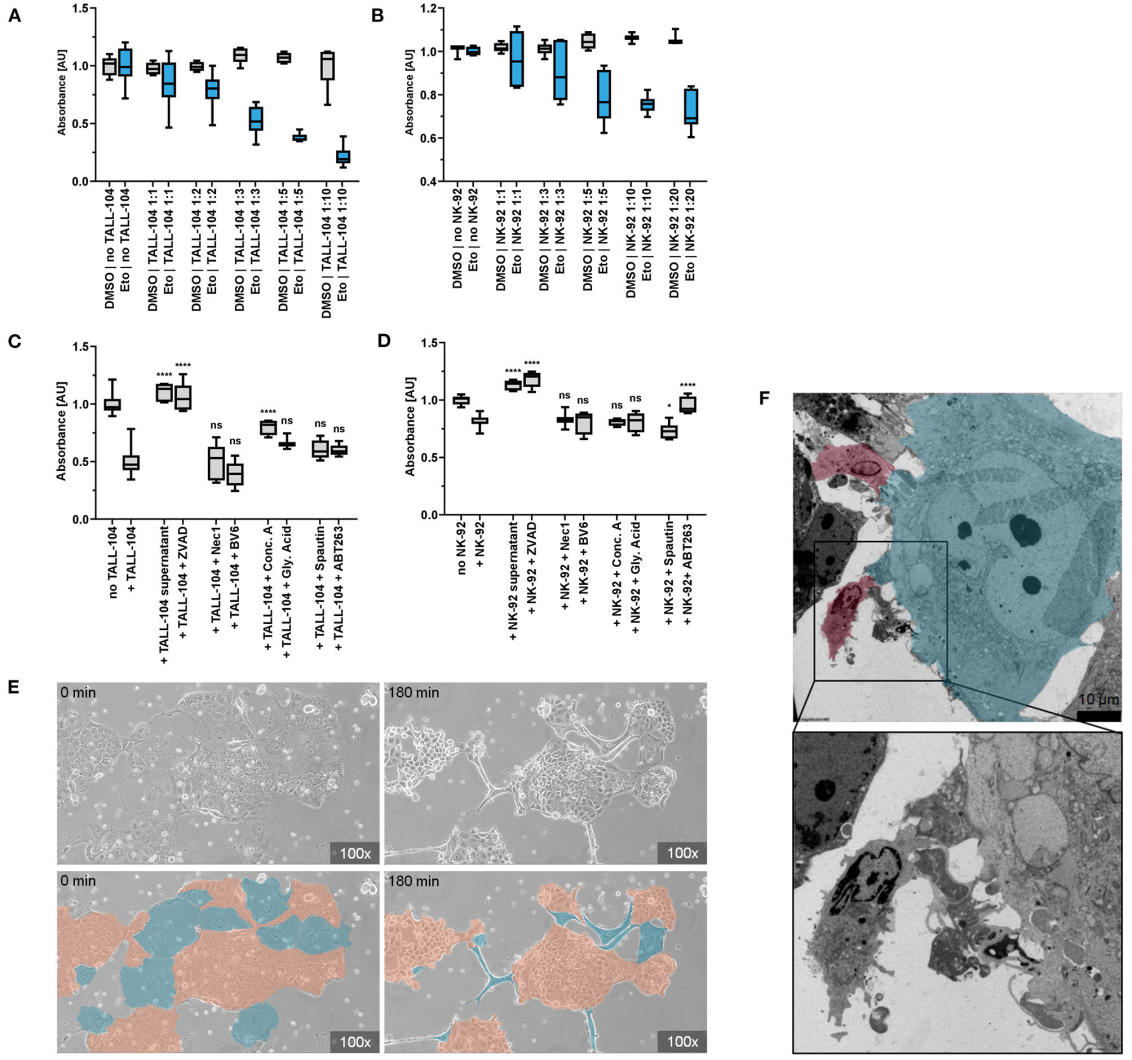
Co-incubation of senescent cells and immune cells. **(A,B)** CRV absorbance of remaining adherent cells after co-incubation of senescent Caco-2 cells and immune cells. Co-incubation with TALL-104 **(A)** or NK-92 **(B)** immune cells lead to dose-dependent elimination of senescent cells. **(C,D)** Elimination of senescent cells under the influence of immune cell conditioned supernatant and various inhibitors of cellular clearance mechanisms as measured by CRV absorbance. TALL-104 **(C)** and NK-92 **(D)** experiments are shown. *p ≤ 0.05, **p ≤ 0.01, ***p ≤ 0.001, ****p ≤ 0.0001, ns = not significant. **(E)** Life cell imaging of co-incubation of senescent cells and immune cells. Specific elimination of senescent cells (blue) by NK-92 cells while proliferating cells (orange) are spared. **(F)** Electron microscopy. Cell-cell contact of TALL-104 cells (red) and a senescent cell (blue).

To explore the senescence-induced immunosurveillance of colonic cancer cells in depth, we conducted a series of co-culture experiments. After 2 h of co-incubation, the number of adherent senescent Caco-2 cells decreased depending on the ratio of immune cells that was added. Addition of NK-92 (displaying properties of natural killer cells) or TALL-104 cells (displaying properties of both cytotoxic T cells and NKT cells) to Caco-2 cells lead to dose-dependent detaching of adherent Caco-2 cells and cell death in >75 % of senescent cells but <20 % of proliferating control cells which was confirmed by CRV staining. This dose-dependent cytotoxicity was not observed in the control group of proliferating cells that had not been exposed to Etoposide (Figures 4A,B). To discern whether this specific elimination of senescent cells was facilitated via factors secreted into the growth medium by the immune cells, we incubated senescent Caco-2 cells with immune cell supernatant. Importantly, addition of conditioned supernatant of TALL-104 or NK-92 cells to Etoposide-pre-treated Caco-2 cells did not decrease cell viability measured by CRV absorption (Figures 4C,D). To confirm the hypothesis that direct cell-cell contact with immune cells accounts for the cell death of senescent Caco-2 cells and to visualize this interaction, we conducted electron microscopy and live cell imaging during co-incubation. Live cell imaging proves directed movement of immune cells toward senescent cells followed by detaching of senescent cells. Non-senescent cells in the environment of senescent cells were not eliminated by the immune cells to the same extent. Electron microscopy of the co-culture experiments shows direct cell-cell contact between TALL-104 cells and senescent Caco-2 cells (Figures 4E,F, Supplementary Video 1).

To determine how immune cells execute the elimination of senescent cells, a set of co-culture experiments was conducted under inhibition of different pathways of cell death. By adding inhibitors of apoptosis, granule exocytosis and necroptosis, the relevance of those pathways for immune cell-mediated elimination was determined. ZVAD has been demonstrated to decrease death receptor mediated cell death in senescent cells (46). Previous studies had not found an impact of caspase-dependent apoptosis on NK cell-mediated senolysis (46). However, we found that using pan-caspase inhibitor ZVAD to block death-receptor-mediated apoptosis resulted in significantly higher quantity of remaining adherent senescent cells after co-incubation resulting from abrogated senolysis of both NK-92 and TALL-104 cells (*p* < 0.0001). Addition of the SMAC-mimetic and apoptosis-sensitizer BV6 however did not have a measurable effect. Inhibiting the necroptosis pathway using Necrostatin-1, an allosteric inhibitor of RIP1, did not reverse the cytotoxicity of TALL-104 or NK-92 cells, suggesting a necroptosis-independent mechanism responsible for the targeted elimination of senescent cells. To assess the role of granule exocytosis for the immune-mediated depletion of senescent cells, we conducted a set of experiments in the presence of Concanamycin A (Conc A) which inhibits perforin-based cytolytic activity by inhibition of vacuolar type H^+^-ATPase. Conc A decreased the cytotoxic effect of TALL-104 cells (*p* < 0.0001) but did not significantly prevent killing of senescent cells by NK-92 cells. HMGB1-Inhibititor glycyrrhizinic acid (Gly. Acid) was used to address HMGB1-dependent metabolic cell death. Spautin-1 (*p* < 0.0230) was used to address autophagy-associated cell death mechanisms. ABT263 partly abrogated the cytotoxic effect of NK-92 cells (*p* < 0.0001) but did not significantly prevent killing of senescent cells by TALL-104 cells. Altogether, our co-incubation, electron microscopy and live cell imaging results indicate that direct cell-cell contact between immune cells and targeted senescent cells is a key mechanism for immune-cell-mediated senolysis. In the presence of inhibitors of apoptosis or (to some extent) granule exocytosis, immune cell-mediated elimination of senescent cells is decreased, suggesting that killing of senescent cells is mainly facilitated via apoptosis induction and via induction of granule exocytosis (Figures 4C,D).

## Discussion

Arresting the cell cycle of premalignant cells as a response to oncogenic signaling and DNA impairment strongly supports the idea of senescence as a beneficial anti-cancer-mechanism (55–57). A premalignant cell's ability to senesce involves major tumor-suppressor pathways and has been proven crucial to fight neoplastic transformation *in vivo* (6, 9, 58–60) and affects treatment outcome of cancer patients (61, 62). Recent studies point to a crucial role of cellular senescence in gastrointestinal diseases including colorectal carcinogenesis (40). Studies report that oncogene-induced senescence (OIS) prevents progression of benign KRAS-mutated sessile serrated adenomas to invasive carcinomas and provides an important barrier opposing malignancy in these early lesions. Malignant transformation to serrated adenocarcinoma requires overcoming this OIS-facilitated cell cycle arrest by downregulation of p16Ink4a (49). There is evidence to suggest that senescence detection might be of predictive value for CRC patients (50), however an extensive clinical study to evaluate the prognostic potential of senescence markers had been missing.

Our study reflects the important and complex role of cellular senescence in colorectal carcinogenesis. In many previous studies, cellular senescence has been described as a “double-edged sword” (34), referring to both the pro- and antitumorigenic effects senescent cells do have on disease progression. We demonstrate that absence of intratumoral senescence and therefore the lack of a basic antitumor defense mechanism is linked with a negative prognosis. Regarding the expression of p21, NTAL, EBP50 and ARMCX3, our results show the important role of senescence induction in tumor defense and underline the relevance of cell cycle regulator p21 and p21-mediated senescence. Moreover, we show that the occurrence of extremely high percentages of senescent cells in CRC is linked to a negative prognosis when compared to patients with moderate expression of senescence markers. These findings point to a complex role of cellular senescence in CRC, suggesting that both non-existent and extensive detection of senescent cells correlate with a negative prognosis. However, further analyses in the context of various molecular and disease subtypes are also necessary to validate our findings.

Senescent cells within the tumor do not automatically imply an effective tumor defense. An effect contributing to the negative outcome of those patients with large numbers of senescent cells might be the inflammatory micromilieu developing in close distance to senescent cells as a driver of further cell damage and therefore accelerating disease progression. While some of the factors secreted after SASP initiation contribute to maintaining the cell cycle arrest and reinforce the senescence program in premalignant cells (63–66) the SASP-driven proinflammatory alterations to the micromilieu–despite enhancing immune surveillance –do as well have malignancy-promoting effects (67).

Secreted factors may provide protumorigenic conditions and stimulate growth, dedifferentiation, and invasiveness of premalignant epithelial cells (68–70). Senescent fibroblasts signaling might contribute to growth-enabling changes to the microenvironment of dormant metastases (71). Increased VEGF expression by senescent cells increases angiogenesis in lesions at risk of malignant transformation and facilitates tumor vascularization, hereby contributing to malignant transformation (72). In CRC, VEGFR2 signaling silences the tumor-antagonizing effect of cellular senescence by actively bypassing p21 (73). There is evidence that the SASP-mediated inflammatory response enhances immune control of senescent tumorous lesions in colorectal carcinoma and prevents malignant transformation in the presence of functional p53 but is protumorigenic in p21/p53-deficient lesions (74).

The perception of senescence as a beneficial anticancer mechanism (55–57) depends on the ability of the immune system to clear senescent cells and prevent negative effects mediated by senescent cells that remain in the tissue (39). Disruption of the tumor immunosurveillance results in accumulation of senescent cells (39), which might be the cause for the negative prognosis we observed in patients with extensive expression of senescence markers. Our study demonstrates that a dichotomous classification does not apply when describing the impact of cellular senescence detection on CRC prognosis. Senescence-associated molecules do have significant prognostic value concerning the outcome of patients with CRC. Moreover, we demonstrate that immune cells *in vitro* specifically eliminate senescent colon cancer cells while somewhat sparing proliferating cells. As Sagiv et al. showed for liver fibrosis in mice (46), we could demonstrate that NK cell-mediated clearance of senescent colorectal carcinoma cells is dependent on granule exocytosis. However, in contrast to Sagiv et al. (46) we found that suppression of the death receptor pathway by ZVAD abrogated the immune cell-mediated elimination of senescent cells in both the NK cell and the cytotoxic T cell model. Thus, our findings lead to the conclusion that induction of both apoptosis and granule exocytosis contribute to the targeted elimination of senescent cells by the immune system.

To reflect this interaction in the clinical setting, proximity analyses of the spatial relation of senescent tumor cells and immune cells are of prognostic relevance and could constitute a prognostic tool in colorectal cancer. Interestingly, we found that the spatial relation of p21-positive tumor cells and cytotoxic T cells is indicative of prognosis regarding DSS and PFS of CRC patients. There is evidence for an immune-infiltration-preventing effect of SASP signaling under certain circumstances (75). High percentage of senescent cells within the tumor–indicative of a negative prognosis as demonstrated in our study–might be the result of impeded tumor immune infiltration due to SASP signaling by senescent cells inhibiting immune cells (75). Furthermore, we showed that patients with close distance of senescent tumor cells and cytotoxic T cells do have a significant better survival which might indicate an antitumorigenic, preferable SASP signaling in these patients. How to impact SASP and induce the preferable, immunosurveillance-promoting secretory activity in senescent cells needs further evaluation and bares great potential in future therapy development. There is *in vitro* (18, 76, 77) and *in vivo* (78–80) evidence for a therapeutic approach inducing cellular senescence in cancerous lesions, evoking immune-cell mediated elimination of cancer cells and enhancing tumor surveillance (41, 44, 81). There are first therapeutic approaches of altering the senescence-induced immune response to induce an antitumorigenic microenvironment (51). Furthermore, therapeutic agents specifically eliminating senescent cells, called senolytics, have demonstrated great potential in various age-associated diseases, including cancer (82).

Taken together, cellular senescence is a key mechanism in opposing malignant transformation of impaired cells. The antitumorigenic effect of cellular senescence is dependent on an intact immune surveillance of the lesion. Therefore, the interaction of immune cells and senescent cells within the tumor microenvironment is of crucial prognostic relevance and provides targets for CRC therapy.

## Data Availability Statement

Datasets presented in this article are not readily available because of the General Data Protection Regulation regarding patient data. Requests to access the datasets should be directed to sebastian.foersch@unimedizin-mainz.de.

## Ethics Statement

The studies involving human participants were reviewed and approved by the Ethical Committee of the Medical Association of the State of Rhineland-Palatinate [ref. no. 837.075.16 (10394)]. Written informed consent for participation was not required for this study in accordance with the national legislation and the institutional requirements.

## Author Contributions

FK and SF: conception and design. FK, SF, AF, and AH: acquisition and data. FK, SF, BK, KT, and MJ: analysis, interpretation of data, and critical revision of the manuscript. FK, SF, and KT: drafting of the manuscript. FK, SF, and MS: statistical analysis. SF: obtaining funding, administrative, and technical, or material support. SF and WR: supervision.

## Funding

This work was supported by the Stage-I-Program of the University Medical Center Mainz and the Mainz Research School of Translational Biomedicine (TransMed).

## Conflict of Interest

The authors declare that the research was conducted in the absence of any commercial or financial relationships that could be construed as a potential conflict of interest.

## Publisher's Note

All claims expressed in this article are solely those of the authors and do not necessarily represent those of their affiliated organizations, or those of the publisher, the editors and the reviewers. Any product that may be evaluated in this article, or claim that may be made by its manufacturer, is not guaranteed or endorsed by the publisher.

## Supplementary Material

Supplementary Figure 1 Additional Kaplan-Meier curves showing **(A)** disease-specific survival with a two-tier cutoff and **(B)** complementary progression-free survival. Cutoffs are displayed as bar graphs and were calculated using a modification of the Charité Cutoff Finder from (54).

Supplementary Table 1 Antibodies and inhibitors used in this study.

Supplementary Table 2 Clinicopathological characteristics of the patient cohort.

Supplementary Table 3 Cutoff values for survival analysis as calculated using a modification of the Cutoff Finder from (54).

Supplementary Video 1 Elimination of senescent Caco-2 cells by TALL-104 immune cells.
