# Supplementary figures and images for: Senescence-Associated Molecules and Tumor-Immune-Interactions as Prognostic Biomarkers in Colorectal Cancer
Source: Front Med (Lausanne). 2022 Apr 12;9:865230. doi: 10.3389/fmed.2022.865230 (PMC9039237; doi:10.3389/fmed.2022.865230)

Supplementary Figure 1

A

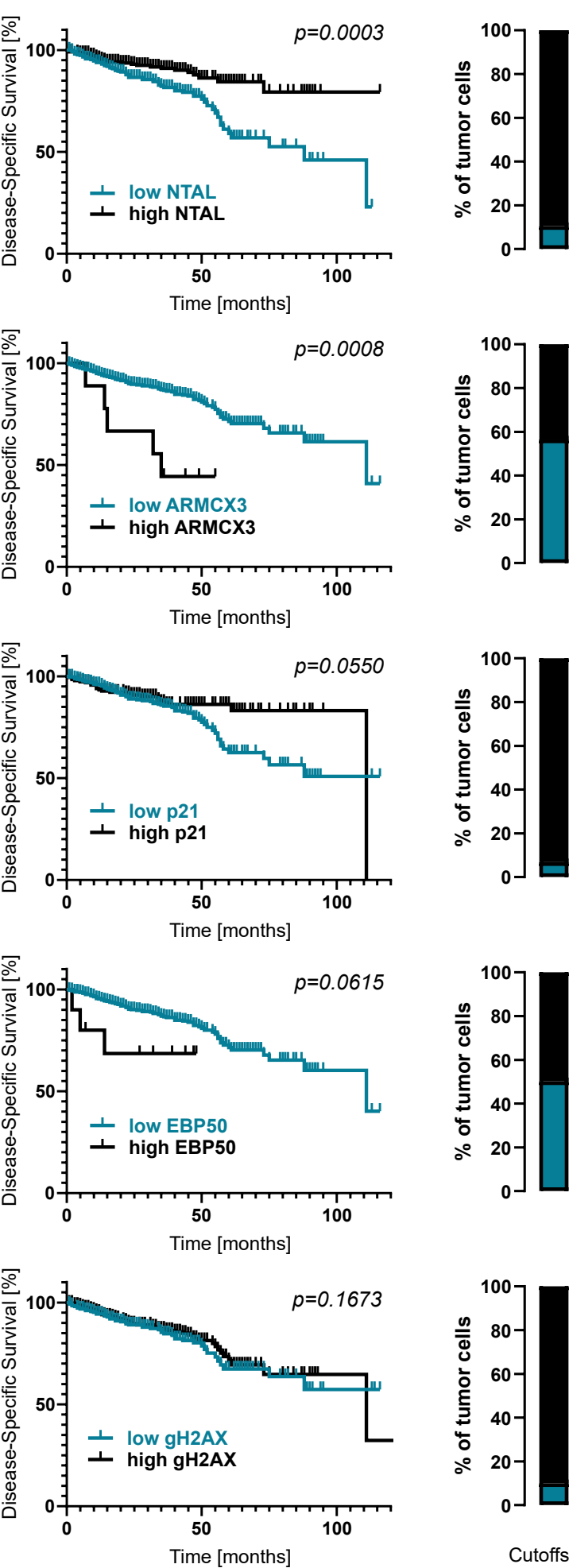

B

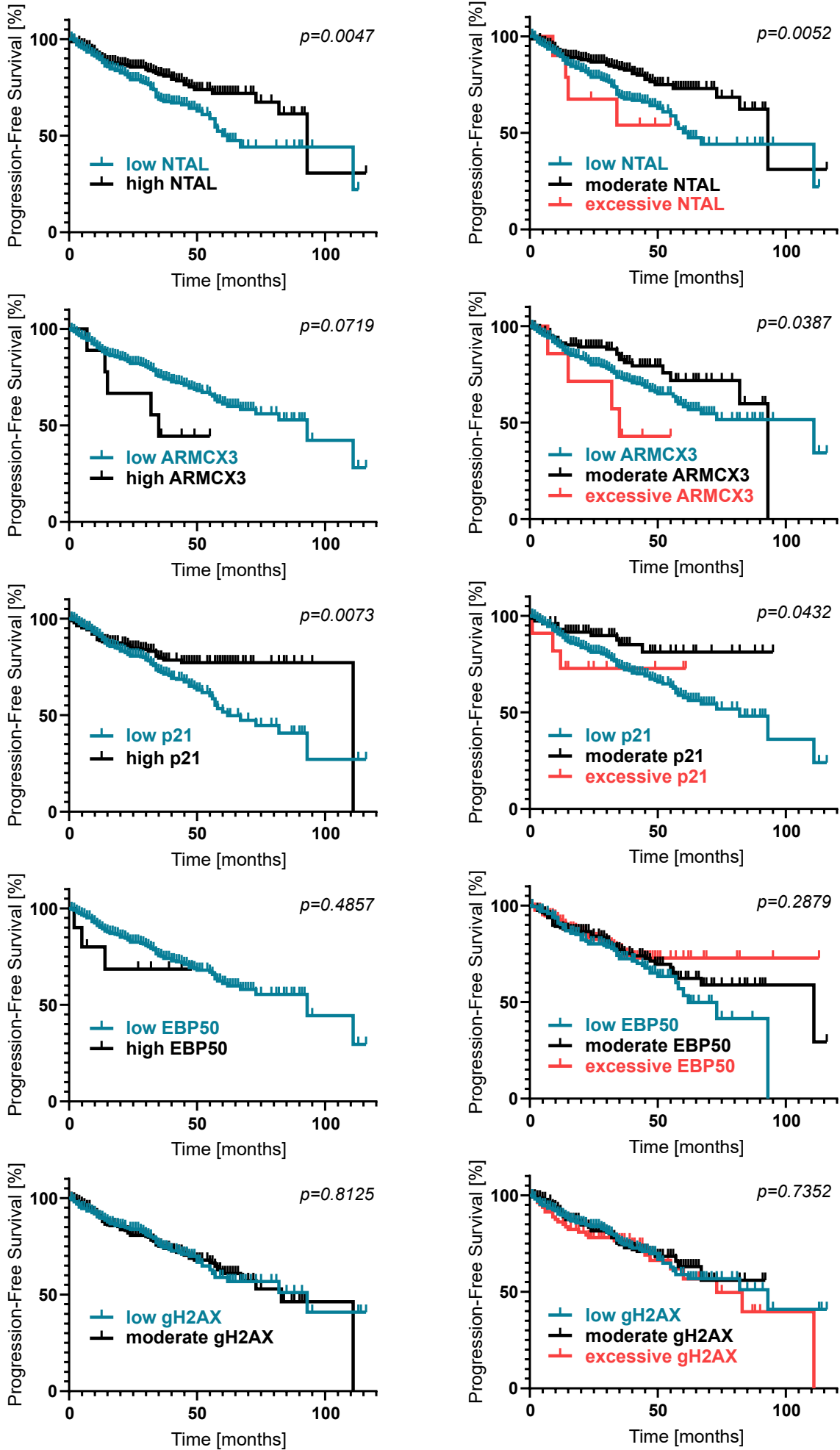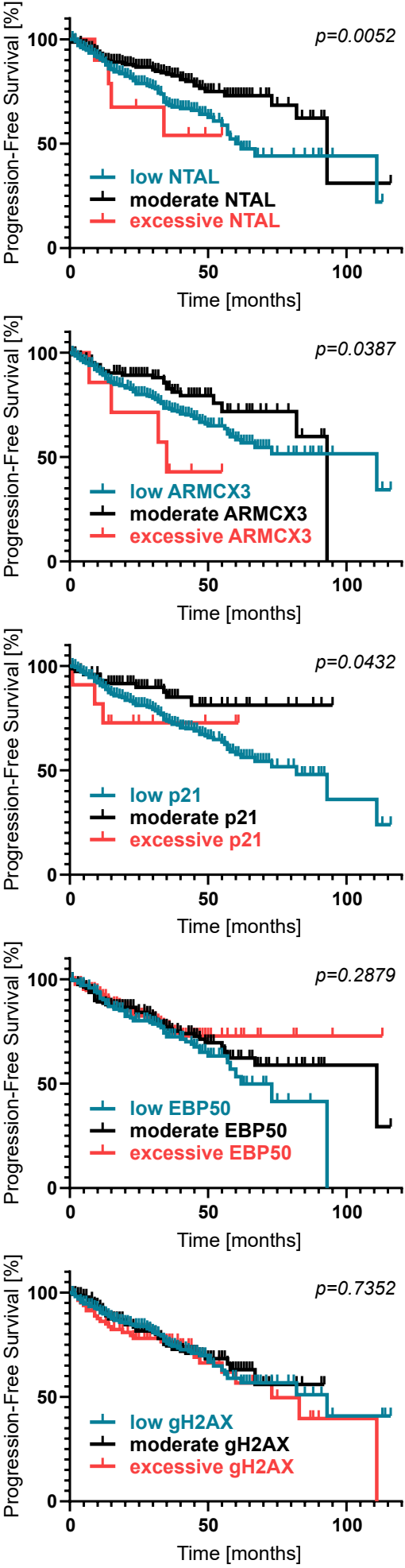

Supplement: Supplementary Figure 1 — Additional Kaplan-Meier curves showing (A) disease-specific survival with a two-tier cutoff and (B) complementary progression-free survival. Cutoffs are displayed as bar graphs and were calculated using a modification of the Charité Cutoff Finder from (54). [file Image_1.pdf]
