# Supplementary material for: Senescence-Associated Molecules and Tumor-Immune-Interactions as Prognostic Biomarkers in Colorectal Cancer
Source: Front Med (Lausanne). 2022 Apr 12;9:865230. doi: 10.3389/fmed.2022.865230 (PMC9039237; doi:10.3389/fmed.2022.865230)
Supplement: Supplementary Table 1 — Antibodies and inhibitors used in this study. [file Table_1.pdf]

Supplementary Table 1

Type	Concentration	Vendor
Inhibitors		
Glycyrrhizinic acid	200 µM	Selleckchem 1405-86-3
Navitoclax ABT263	1,25 µM	Selleckchem 923564-51-6
Spautin-1	10 µM	Sigma SML0440-5MG
Concanamycin	50 µM	Selleckchem S7023
Necrostatin-1	20 µM	Sigma N9037-10MG
Antibodies		
anti ARMCX3	1:200	Abcam ab98938
anti p21	1:50	Dako Clone SX118
anti EBP50	1:100	Abcam ab109430
anti NTAL	1:50	Abcam ab3992
anti gH2AX	1:150	Cell Signaling #9718
