# Supplementary material for: Senescence-Associated Molecules and Tumor-Immune-Interactions as Prognostic Biomarkers in Colorectal Cancer
Source: Front Med (Lausanne). 2022 Apr 12;9:865230. doi: 10.3389/fmed.2022.865230 (PMC9039237; doi:10.3389/fmed.2022.865230)
Supplement: Supplementary Table 2 — Clinicopathological characteristics of the patient cohort. [file Table_2.pdf]

Supplementary Table 2

N =	598
Sex	
male	323
female	275
Age (years)	
average	70.3
T-Stage	
T1	52
T2	121
T3	371
T4	54
N-Stage	
N0	345
N1	153
N2	88
NX	12
M-Stage	
M0	317
M1	61
MX	220
UICC	
I	135
II	210
III	192
IV	61
Grading	
G1	7
G2	429
G3	162
