# Supplementary material for: Senescence-Associated Molecules and Tumor-Immune-Interactions as Prognostic Biomarkers in Colorectal Cancer
Source: Front Med (Lausanne). 2022 Apr 12;9:865230. doi: 10.3389/fmed.2022.865230 (PMC9039237; doi:10.3389/fmed.2022.865230)
Supplement: Supplementary Table 3 — Cutoff values for survival analysis as calculated using a modification of the Cutoff Finder from (54). [file Table_3.pdf]

Supplementary Table 3

Marker	low	high	low	moderate	high
NTAL	< 10.52	> 10.52	< 11	11 - 63	> 63
ARMCX3	< 56.45	> 56.45	< 17.5	17.5 - 63	> 63
p21	< 6.83	> 6.83	< 15	15 - 38	> 38
EBP50	< 50.2131	> 50.2131	< 0.81	0.81 - 6.19	> 6.19
gH2AX	< 10*	> 10*	< 2*	2 - 10*	> 10*

*positive cells per core

p21 | CD8

average distance		CD8+ within 100 µm of p21+	
close	far	low	high
< 145 µm	> 145 µm	< 50 %	> 50 %
